# Biosensors Based on Isothermal DNA Amplification for Bacterial Detection in Food Safety and Environmental Monitoring

**DOI:** 10.3390/s21020602

**Published:** 2021-01-16

**Authors:** Sandra Leonardo, Anna Toldrà, Mònica Campàs

**Affiliations:** 1 Institut de Recerca i Tecnologies Agroalimentàries (IRTA), Ctra. Poble Nou km 5.5, Sant Carles de la Ràpita, 43540 Tarragona, Spain; sandra.leonardo@irta.cat; 2 Department of Fibre and Polymer Technology, KTH Royal Institute of Technology, Teknikringen 56, 10044 Stockholm, Sweden; anntf@kth.se

**Keywords:** bacteria, biosensor, isothermal DNA amplification, food safety, environmental monitoring, loop mediated isothermal amplification (LAMP), rolling circle amplification (RCA), recombinase polymerase amplification (RPA), helicase dependent amplification (HDA), strand displacement amplification (SDA), isothermal strand displacement polymerisation (ISDPR)

## Abstract

The easy and rapid spread of bacterial contamination and the risk it poses to human health makes evident the need for analytical methods alternative to conventional time-consuming laboratory-based techniques for bacterial detection. To tackle this demand, biosensors based on isothermal DNA amplification methods have emerged, which avoid the need for thermal cycling, thus facilitating their integration into small and low-cost devices for in situ monitoring. This review focuses on the breakthroughs made on biosensors based on isothermal amplification methods for the detection of bacteria in the field of food safety and environmental monitoring. Optical and electrochemical biosensors based on loop mediated isothermal amplification (LAMP), rolling circle amplification (RCA), recombinase polymerase amplification (RPA), helicase dependent amplification (HDA), strand displacement amplification (SDA), and isothermal strand displacement polymerisation (ISDPR) are described, and an overview of their current advantages and limitations is provided. Although further efforts are required to harness the potential of these emerging analytical techniques, the coalescence of the different isothermal amplification techniques with the wide variety of biosensing detection strategies provides multiple possibilities for the efficient detection of bacteria far beyond the laboratory bench.

## 1. Introduction

Bacterial contamination of food, water and environment is a crucial safety issue as it is linked to human and animal diseases, some of them leading to death and great economic losses. About 91% of all foodborne diseases are caused by bacterial contamination, being *Escherichia coli*, *Salmonella* and *Campylobacter* the leading causes [1]. The intensified trade in goods and services, the increased population mobility, the climate change, and other factors linked to globalisation make bacterial pathogens spread around the world much faster than ever, making evident the necessity for proper monitoring of pathogens.

Current methods of bacterial detection rely upon laboratory-based techniques such as cell culture, microscopy analysis and biochemical assays [2]. These procedures require specialised equipment and trained users. Microscopy involves staining bacteria and observing their morphology and staining pattern, which is relatively quick but not specific, whereas culturing bacteria on selective media under specific growth conditions can take up to several days. Furthermore, not all bacteria can be cultured in the laboratory. Biochemical assays include enzyme assays for the detection of specific enzymes from bacteria and immunological tests, such as enzyme-linked immunosorbent assays (ELISAs) or agglutination assays usually employed to detect particular surface epitopes. The advent of molecular techniques has enabled more rapid and specific identification of bacterial strains. However, traditional techniques such as polymerase chain reaction (PCR) involve multiple thermocycling steps using high-precision instruments difficult to miniaturise, requiring stringent conditions of laboratory compartmentalisation and thus almost exclusively performed in centralised laboratories, which results in a time lag between sample procurement and analysis, increasing the risk of contamination [3,4].

As a consequence, the need for portable and autonomous systems able to perform accurate and rapid analysis in situ is obvious. The combination of isothermal DNA amplification methods with biosensing strategies responds to this demand. Biosensors are bioanalytical tools that incorporate a biorecognition element in intimate contact with a physico-chemical transducer [5]. While the bioreceptor recognises the target analyte, the transducer converts the biorecognition event into a measurable signal, which can be electrochemical, optical, piezoelectric or thermal. Ideally, for a biosensor to be useful, it should be sensitive, selective, precise, fast, cheap, miniaturised and automated. Isothermal DNA amplification techniques can contribute to this objective. Unlike typical thermal cycling amplification, isothermal techniques are carried out at a constant temperature, which reduces the power needed and makes them more compatible for integration into miniaturised systems [4]. These techniques have provided biosensors with the relevant sensitivity and specificity for the detection of different analytes such as microalgae [6,7] viruses [8,9], fungi [10,11], protozoa [12,13] and bacteria, being the latter the focus of this review. Therefore, the objective of this work is to provide a comprehensive overview of the biosensors based on isothermal DNA amplification methods for bacterial detection with application in the fields of food safety and environmental monitoring, ultimately affecting human, animal and plant health. It also faces the key factors that are crucial in the development of these biosensors and the advantages and limitations of the use of isothermal DNA amplification methods.

## 2. Isothermal DNA Amplification-Based Biosensors: State of the Art

Optical and electrochemical biosensors based on loop-mediated isothermal amplification (LAMP), rolling circle amplification (RCA), recombinase polymerase amplification (RPA), helicase dependent amplification (HDA), strand displacement amplification (SDA), and isothermal strand displacement polymerisation (ISDPR) are described in next sections and summarised in Table 1. Colorimetric and fluorescent assays as well as lateral flow systems, although widely explored, are not included in this work as they are not proper biosensors. Biosensors based on isothermal RNA amplification techniques (e.g., nucleic acid sequence-based amplification (NASBA)) or those including a reverse transcription step are not included either.

### 2.1. LAMP-Based Biosensors

**Figure 1:**
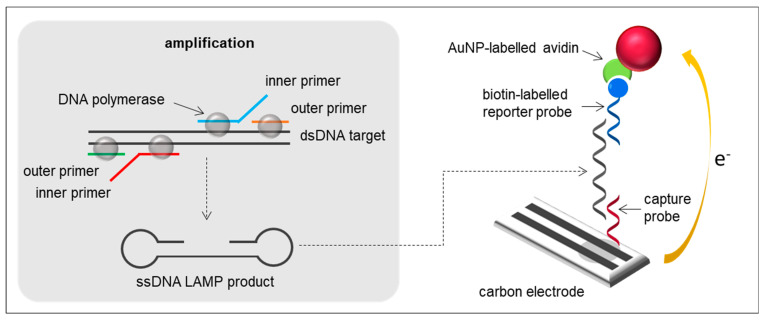
LAMP principle and example of a LAMP-based electrochemical biosensor using a capture probe, a biotin-labelled reporter probe, AuNP-labelled avidin and a carbon electrode.

LAMP is based on auto-cycling strand displacement DNA synthesis carried out by *Bacillus stearothermophilus* (*Bst*) DNA polymerase large fragment under isothermal conditions between 60 and 65 °C for around 60 min [42,43]. In LAMP, four primers, comprising two inner primers and two outer primers, are used to recognise six specific regions of target DNA. One inner and one outer primer are forward primers, while the other two are reverse primers, which form stem-loops motifs with self-priming capability. This approach results in an amplification strategy where the target sequence is copied with each round of replication and remains tethered to the previous amplicon, resulting in cauliflower-like single-stranded DNA (ssDNA) structures bearing multiple loops as well as stem-loop DNAs of different sizes (Figure 1). The use of additional “loop primers”, which bind to loop structures resulting in double-stranded DNA (dsDNA) products, can reduce the reaction times [44]. Although LAMP has been the most widely exploited isothermal DNA amplification method since it was reported by Notomi and co-workers in 2000 [43], most detection strategies are based on real-time detection of LAMP amplicons in solution and fewer are the works that report the development of LAMP-based biosensors for the detection of bacteria. Electrochemical monitoring of amplicon formation in real-time is almost always based on the time-course measurement of the interactions occurring between a redox-active reporter added to the reaction solution and the DNA products or by-products generated during the isothermal amplification. Martin and co-workers used four different redox reporters (i.e., three intercalating reporters with different ds-DNA binding strengths ([Os(bpy)_2_dppz]^2+^, methylene blue and a methylene blue derivative), and one non-intercalating probe able to sense the pyrophosphate ion concentration produced during LAMP (Ru(NH_3_)_6_^3+^), for the real-time electrochemical detection of *Flavobacterium columnare* [45]. *F. columnare* is an important pathogen of freshwater fish causing columnaris disease, which detection is a major concern in the catfish and warm water fish farming industries [46]. Current responses generated from the free-to-diffuse redox mediator in solution were recorded by square wave voltammetry (SWV). As LAMP reaction progressed, an increasing amount of the redox reporter was bound to the exponentially amplified dsDNA amplicons, or in the case of the non-intercalating Ru(NH_3_)_6_^3+^ species, to the exponentially released by-product pyrophosphate anion, resulting in a decrease of the current response due to the lower diffusion coefficient of the bound redox-active probe compared to the free one. Similar approaches have been used for the detection of the *vt* gene in *E. coli* O157:H7 [47,48] and the *invA* gene in *Salmonella* enterica [47,49] using methylene blue, the *Tuf* gene in *E. coli* [50,51] and *Mcat* [51] and *catalase* [50] genes in *Staphylococcus aureus* using osmium [51] and ruthenium redox complexes [50], and *E. coli* [52], *Vibrio parahaemolyticus* [53] and *Mycobacterium tuberculosis* [54] using the redox intercalator Hoechst-33258. 

Regarding LAMP-based biosensors, most strategies perform amplification in solution to avoid steric hindrance effects observed in solid-phase amplification and to provide higher efficiency. Once amplified, products are hybridised with a complementary short sequence called capture probe, which is immobilised on the transducer, in order to be detected.

An optical biosensor for the detection of *femB* and *mecA* genes of methicillin-resistant *S. aureus* (MRSA) was developed by Nawattanapaiboon and co-workers [14]. *S. aureus* has the ability to make seven different toxins that are frequently responsible for Staphylococcal food poisoning [55]. These toxins are fast acting, and patients experience symptoms (nausea, vomiting, stomach cramps and/or diarrhoea) within one to eight hours after eating contaminated food. The most common way for food to be contaminated with *S. aureus* is through contact with food workers who carry the bacteria or through contaminated milk and cheeses. The need for multiple primers in LAMP induces high complexity for multiplex assays in “one spot”. For this reason, duplex amplification of *S. aureus* was carried out in a parallelised way, i.e., performing amplifications separately in single reactions. Specific biotinylated capture probes for each gene were immobilised on a streptavidin-modified chip and the hybridisation process with LAMP amplicons was monitored by surface plasmon resonance (SPR). To increase the sensitivity of the assay, the signal was amplified using a sandwich assay with Au nanoparticle (AuNP)-labelled reporter probes. The use of AuNP-labelled reporter probes increased the sensitivity of the SPR biosensor by one order of magnitude, providing a limit of detection (LOD) of 10 copies/µL. A sandwich format was also adopted for the development of an electrochemical biosensor for the detection of *Vibrio cholerae* [15]. This bacterium is usually found in water or food sources that have been contaminated by faeces from a person infected with cholera [56]. Thus, cholera is most likely to be found and spread in places with inadequate water treatment, poor sanitation, and inadequate hygiene. Although cholera infection is often mild or without symptoms, it can sometimes be severe (diarrhoea, vomiting and leg cramps), and even lead to death within hours if no treatment is undertaken. In this sandwich assay, capture probes were immobilised on screen-printed carbon electrodes and AuNP-labelled reporter probes were detected by differential pulse anodic stripping voltammetry (DPASV) (Figure 1). LAMP products were detected at concentrations as low as 50 ng/µL. In both cases, a 95 °C denaturation step of the dsDNA LAMP amplicons was required for their hybridisation with the capture and reporter probes.

Sun and collaborators [16] reported an electrochemical biosensor for the detection of *Yersinia enterocolitica*. This bacterium causes yersiniosis, an infection usually caused by the consumption of contaminated raw or undercooked pork [57]. Infection occurs most often in children and in the winter, and common symptoms are fever, abdominal pain and diarrhoea. For the development of the biosensor, the capture probe was immobilised on a carbon electrode modified with chitosan/nano-V_2_O_5_/multi-walled carbon nanotubes (MWCNTs). The redox intercalator methylene blue was used to monitor the hybridisation of the ssDNA amplicon to the ssDNA capture probe. Unlike previous works that monitored amplicon formation in solution with methylene blue also in solution, in this work the amplicon was immobilised and thus the redox mediator as well. Increasing amounts of amplified product resulted in higher amounts of redox mediator, thus resulting in a proportional increase of the reduction peak. The synergistic effect of nano-V_2_O_5_ and MWCNTs increased the amount of capture probes immobilised on the electrode surface and subsequently the amount of the amplicon hybridised, enhancing the electrochemical response. The LAMP-based biosensor was applied to the detection of *Y. enterocolitica* in naturally contaminated pork meat samples. Patterson and co-workers [17] developed a LAMP-based electrochemical biosensor for the detection of *S. enterica*. *S. enterica* infection can result in enterocolitis/diarrhoea, bacteraemia, enteric (typhoid) fever and/or chronic asymptomatic carriage. Many serovars infect both humans and animals through consumption of contaminated food, and the severity is a function of the serovar, strain virulence and host susceptibility [58]. Thus, discrimination of *S. enterica* serovars is highly desired. The authors used LAMP primers that amplify a target region in the *recF* gene of most of the *Salmonella* serovars but that produce products that differ significantly in their ssDNA regions. Two different capture probes complementary to these ssDNA regions were used for the discrimination of *S. enterica* subsp. *enterica* serovars Typhimurium and Choleraesuis, the causative agents of enterocolitis and sepsis in humans, respectively. The capture probes were modified with a flexible Letsinger trihexylthiol at their 5′ end for self-assembling on gold surfaces and with a methylene blue redox reporter at their 3′ end. When LAMP amplicons were absent, the flexibility of this ssDNA probe ensured that the terminal redox moiety collided with the electrode surface, generating a redox current that was measured by SWV. Upon hybridisation with the amplicon, the redox moiety was held in a position far away from the electrode surface and a decrease in the SWV signal was observed. The integration of the electrochemical detection system together with microfluidic technology enabled to perform amplification and detection in a single miniaturised platform. On the other hand, Yang and collaborators [18] developed an electrochemical biosensor for the detection of Panton-Valentine Leukocidin (PVL) toxin gene of MRSA. In this case, a biotinylated primer was used in LAMP and the resulting amplicons were bound to avidin-modified AuNPs without the need of a capture probe. A second primer linked to AuNPs through a thiol group was hybridised to the loop regions of the AuNP-labelled amplicons to further increase the assay sensitivity and specificity. The nano-assemblies were subjected to resistive pulse sensing (RPS) analysis and the blockade event produced by the passage of the particles through a membrane containing a tuneable pore was recorded. The system attained an LOD of 530 copies of genomic DNA.

Li and co-workers [19] combined an electrochemical immunosensor for the selective capture and rapid identification of *E. coli* O157:H7 with an optical microfluidic chip-based LAMP. This *E. coli* serogroup produces Shiga toxin, whose infection causes severe stomach cramps, diarrhoea and vomiting. Some infections are very mild and people get better within 5 to 7 days, but others are severe or even life-threatening [59]. In their work, an anti-*E. coli* O157:H7 polyclonal antibody-modified carbon nanotube (CNT) multilayer electrode was used for the selective capture of the target. The use of CNTs increased the surface area, allowing the immobilisation of higher amounts of antibody, and increased the electrical conductivity. Changes in the charge transfer resistance were used to monitor the antigen-antibody interaction. Captured bacteria were then cultured in a specific culture medium to increase the number of *E. coli* O157:H7 cells. DNA from the bacteria was then extracted and analysed using a microfluidic chip-based LAMP coupled with a fiber optic sensor that monitored the turbidity of the solution produced by the white precipitate magnesium pyrophosphate released during the isothermal amplification process. The detection system displayed high specificity and enabled the detection of *E. coli* O157:H7 at concentrations as low as 1 colony-forming unit (CFU)/mL. Recently, a lab-on-a-chip (LOC) device for *S.* Typhimurium detection has been reported [20]. LOC platforms aim at simplifying manual and time-consuming laboratory work combining multiple analytical processes and sample preparation steps in micro platforms with a high degree of automation. In this work, the proposed LOC is based on an oxygen plasma nanotextured polymeric chip combining, in one microfluidic chamber, bacteria immunoaffinity capturing on the chip surface, chemical lysis and LAMP amplification, followed by label-free surface acoustic wave (SAW) detection. The LOC platform with a prior 3 h off-chip pre-culturing and centrifugation step for concentration enabled to detect 1 *S.* Typhimurium cell in 25 mL of milk, fulfilling the legislation requirements [60].

The use from four to six specially designed primers recognising six to eight regions of the target DNA sequence provides LAMP a higher specificity compared to other isothermal amplification methods. Moreover, the auto-cycling reactions lead to the accumulation of large amounts of target DNA and other reaction by-products that allow rapid detection using different formats.

### 2.2. RCA-Based Biosensors

**Figure 2:**
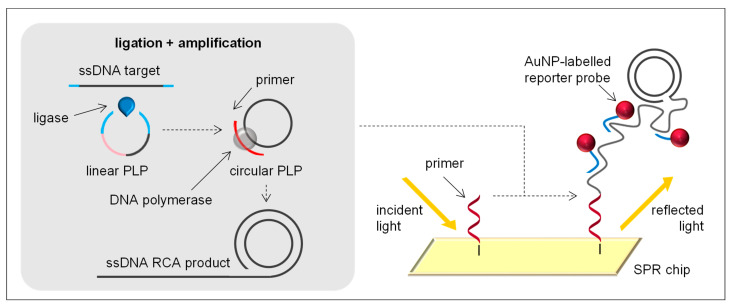
RCA principle and example of an RCA-based SPR biosensor using an immobilised primer, a AuNP-labelled reporter probe and an SPR chip.

RCA is another isothermal enzyme process acting on circular DNA molecules. RCA exploits the strand displacement and highly processive polymerase activity of the Phi29 bacteriophage DNA polymerase (Φ29DNAP) to extend single or multiple primers annealed to a circular DNA template [61]. The strand displacement activity allows the newly synthesised DNA template to displace the previously generated DNA molecule, releasing ssDNA. The requirement of a circular template for amplification makes RCA a leading candidate for amplification of DNA molecules that exist in vivo as circular molecules, such as plasmids and certain phages [62]. Nevertheless, RCA can be performed from non-circular DNA targets using padlock probes (PLPs), yielding circular structures from linear DNA and greatly increasing specificity (Figure 2).

RCA was reported for the first time in 1995 [63] and some years after started to be used in the development of biosensors for the detection of different bacterial pathogens. Their detection required the ligation of the target gene into a PLP. This circularisation process demands a ligase for the specific circularisation of the PLP (with 3′-hydroxy and 5′-phosphate) using the target sequence of the genomic DNA as a template. The hybridisation temperature of the PLPs is an important factor in the ligation reaction and should minimise the second structure of PLP to assure the full hybridisation with the target [21]. Thus, although most works perform RCA at 37–40 °C for around 40 min, longer times and higher temperatures (e.g., 37–95 °C) are required for the target ligation with PLP and exonuclease treatment, increasing the complexity and time of the overall process to around 150 min. Additionally, an initial denaturation step of the genomic dsDNA at 95 °C is also required.

Xiang et al. [22] developed an SPR biosensor method using surface-anchored RCA and AuNP-labelled reporter probes to isothermally detect multiple point mutations associated with drug resistance in *M. tuberculosis*. This bacterium usually attacks the lungs and cause tuberculosis. Infection is usually spread through the air from one person to another [64]. In the development of the biosensor, different PLPs containing a target-complementary sequence, a general sequence, and a tag sequence complementary to the specific primers immobilised on different channels of the SPR chips, were used. Upon recognition of the point mutation on DNA targets, PLPs were circularised by ligation and hybridised to the corresponding immobilised primers. Primer-PLP complexes were isothermally amplified by RCA, and AuNP-labelled reporter probes were hybridised to RCA products for signal amplification (Figure 2). Although amplification was performed on solid phase, the ligation of the target DNA with the PLP was performed in solution, which showed better specificity and efficiency in mutation discrimination than chip-based ligase-mediated RCA [65]. Following a similar strategy, solid-phase RCA and SPR were combined for the multiplex detection of *E. coli*, *Shigella dysenteriae*, *Staphylococcus epidermis*, *S. aureus*, *Streptococcus pneumoniae* and *Enterococcus faecalis* [23]. Biotinylated primers were immobilised on streptavidin-AuNPs, which were previously bound to biotin-labelled secondary antibodies spotted on the chip surface through biotin binding with one of the four binding sites of streptavidin. RCA was performed on the AuNPs after PLP ligation with the target and hybridisation with the primer. AuNP-labelled reporter probes were hybridised to RCA amplicons and changes in the refractive index of the SPR biosensor were recorded, achieving an LOD as low as 0.5 pg/µL of genomic DNA. In another work, Xiang and co-workers [21] performed RCA in solution and implemented a cleavage reaction to produce small fragments of ssDNA, which immediately hybridised with the capture probe immobilised on AuNPs assembled onto the SPR chip surface. This strategy enabled the real-time monitoring of *M. tuberculosis* and *Mycobacterium avium* complexes. Whereas *M. tuberculosis* is usually transmitted through the air, *M. avium* has been found in water, dust and soil, but people are also infected when the bacterium is inhaled or swallowed [66]. The optical biosensor attained LODs of 5 pg/µL and 2 pg/µL of genomic DNA for *M. tuberculosis* and *M. avium*, respectively.

Although electrochemistry is the detection technique most commonly used in biosensors, only one work has been found that combines RCA with this technique. An RCA-based electrochemical biosensor for the detection of *Clostridium tetani*, the causative agent of tetanus disease, was reported by the immobilisation of capture probes on MWCNT-AuNP-modified electrodes [24]. After hybridisation of RCA amplicons with the capture probes, a sandwich configuration was adopted using methylene blue-labelled reporter probes and DPV as a detection method.

Less commonly used detection techniques such as ferromagnetic resonance (FMR) and terahertz (THz) spectroscopy have also been exploited in isothermal DNA amplification systems, in this case for the detection of *V. cholerae* and *E. coli*, respectively. For *V. cholerae* detection, magnetic nanoparticle (MNP)-labelled reporter probes were used [25]. Hybridisation of the MNP-labelled reporter probes to RCA amplicons resulted in the aggregation of MNPs, which lead to a shift of the FRM spectra. For *E. coli* detection, primers were immobilised on magnetic beads (MBs) leading to RCA reaction upon recognition of the circularised PLPs [26]. RCA products were separated from complex matrix components by the application of a magnetic field. As DNA molecules are less absorptive than water molecules in the THz range, the RCA products on the surface of the MBs caused a significant decrease in THz absorption.

In addition to the linear RCA kinetics originally described, hyperbranched RCA (HRCA) has been used to increase the amplification power. This method employs secondary primers that target the amplification product [61]. As the initial ssDNA product is elongated from the circular DNA, these supplementary primers bind at regular intervals along the repeating strand and initiate additional primer elongation events. The multiple elongating strands displace downstream strands resulting in further exposed sites for primer binding, originating a hyperbranched, self-propagating pattern of primer extension and strand displacement. Long and co-workers [27] developed a biosensor for *L. monocytogenes*, a bacterium that causes listeriosis, a very dangerous infection for foetus or new-born babies [67]. The authors combined solid-phase HRCA as a DNA amplification method, MBs as immobilisation supports, and electrochemiluminescence (ECL) as a detection method. A tris(bipyridine)ruthenium (TBR)-labelled primer was used for ECL detection, a type of luminescence produced by electrochemical oxidation or reduction that integrates both the advantages of electrochemistry and spectroscopy, such as avoiding background signals from scattered light and luminescent impurities, and without requiring a light source. *L. monocytogenes* was detected at levels as low as 0.2 pg/µL of genomic DNA with high specificity.

RCA allows the amplification of both linear and circular DNA, resulting in concatenated ssDNA, or dsDNA when HRCA is performed, with a linear configuration that is usually detected using labelled-primers or reporter probes. Amplicon cleavage has been explored to produce a large number of small ssDNA fragments from one amplicon, resulting in signal amplification. The need for PLP ligation of linear target DNA to yield circular structures requires longer times of the overall process compared to other isothermal amplification methods and usually requires changes in the operating temperature, which limits the advantages of isothermal amplification methods in the development of biosensors.

### 2.3. RPA-Based Biosensors

**Figure 3:**
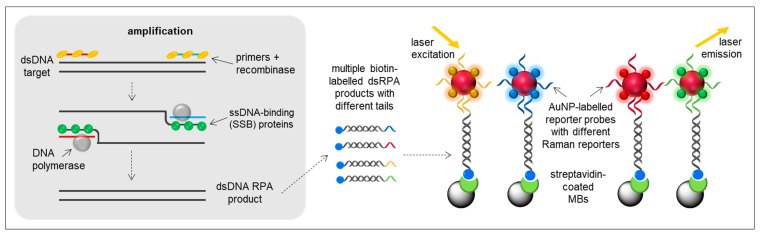
RPA Principle and example of an RPA-based SERS platform using biotin-labelled RPA products with different tails, AuNP-labelled reporter probes with different Raman reporters and streptavidin-coated MBs.

RPA is becoming a molecular tool of choice for rapid, specific, and cost-effective identification of pathogens. First reported by Piepenburg and collaborators in 2006 [68], the RPA process employs three enzymes: a recombinase, a ssDNA-binding protein (SSB) and a strand-displacing polymerase. While the recombinase matches oligonucleotide primers with their homologous sequences in the target DNA, SSB binds to the displaced strand of DNA and prevents the dissociation of primers. Finally, the strand-displacing polymerase begins DNA synthesis where the primer has bound to the target DNA (Figure 3). With the use of two primers, exponential amplification of the target sequence is achieved at a constant temperature of around 37–40 °C in 20–40 min.

One important factor to take into account to favour diverse downstream RPA applications is the incorporation of labels into the nucleic acid amplicon during RPA reaction to allow the capture and/or detection of the amplicons in the subsequent assays. Nucleic acid labelling can be achieved terminally using labelled primers or internally through labelled nucleotides. Terminal labelling is performed to the 5′-end of primers so that they can be captured and detected by the corresponding recognition molecules and the 3′-end of the DNA strand is available to allow DNA polymerases to incorporate free nucleotides. Terminal labelling can be a good choice when the downstream application is a sandwich assay, as the two labels incorporated via terminal labelling are further apart from each other, separated by the amplicon. This distance in terminal labelling may prevent steric hindrance compared to internal labelling. RPA sandwich configurations have been adopted in the development of biosensors for *Piscirickettsia salmonis* [28], *Franciscella tularensis* [29,30], *Salmonella* spp. [31], *Pseudomonas syringae* [32], *Botrytis cinarea* [33] and *Fusarium oxyspurum* [33].

Sabaté del Río and co-workers [28] developed an electrochemical biosensor for the detection of *P. salmonis*, a bacterium that causes piscirickettsiosis disease in salmonid fishes. Thiolated primers were self-assembled on gold electrodes, and solid-phase RPA was achieved in the presence of the specific target sequence and biotinylated reverse primers. The formation of the subsequent surface-tethered duplex amplicons was electrochemically monitored by the addition of streptavidin-horseradish peroxidase (HRP) conjugate. The HRP enzyme catalyses the oxidation of 3,3′,5,5′-tetramethylbenzidine (TMB) in the presence of H_2_O_2_, for the subsequent detection of oxidised TMB. The solid-phase RPA approach allows integrating DNA amplification, hybridisation and detection on a platform, thus reducing analysis time and contamination, as well as paving the road to on-site testing. However, the immobilisation of the primers on a solid support leads to lower amplification yields than in solution, mainly due to the steric hindrance, which negatively impacts the enzyme efficiency during amplification. To alleviate this issue, the incorporation of a polyT spacer in the immobilised primer is highly recommended. In a subsequent work, Sabaté del Río and co-workers [29] addressed the optimisation of vertical but also lateral spacing of immobilised primers in order to obtain maximum signal-to-noise ratios and lower LODs. They also evaluated the use of HRP-labelled primers to avoid one incubation step and thus simplify the assay. The optimised protocol was successfully applied to the detection of *F. tularensis*, the causative agent of tularaemia, the pneumonic form of which is often lethal without treatment. The biosensor attained an LOD of 5.3 fM of synthetic DNA, which represents two orders of magnitude lower than when using the non-optimised surface and biotin-labelled primers. Instead of employing biotinylated primers, Sánchez-Salcedo and collaborators [31] used FITC-labelled primers in the RPA in solid phase, followed by incubation with an HRP-labelled anti-FITC antibody conjugate to detect *Salmonella* spp.

Solid-phase RPA has also been coupled with optical detection techniques for the determination of *F. tularensis* [30]. *F. tularensis* forward primers were immobilised on a ring resonator surface and changes in the resonant wavelength were measured, enabling label-free and real-time monitoring of DNA amplification. Although the LOD achieved by the optical biosensor was two orders of magnitude higher compared to the beforementioned electrochemical biosensor [29], the label-free format greatly reduced the time and complexity of the assay.

Nowadays, innovation hinges on the use of tailed primers (primers modified with short oligonucleotide tails) that result in dsDNA products flanked by ssDNA tails. Lau and co-workers explored the use of tailed primers for the development of an electrochemical biosensor for the detection of *P. syringae* [32] and an optical biosensor for the multiplexed detection of *P. syringae*, *B. cinerea* and *F. oxysporum* [33], all of them plant pathogens. In the electrochemical biosensor, a 5′-tailed forward primer and a biotin-labelled reverse primer were used for RPA amplification in solution [32]. The amplicons were hybridised with AuNP-capture probes and bound to streptavidin-MBs. In order to prevent the interference in the electrochemical signal from iron ions of the MBs, the conjugates were heat-treated at 95 °C to denature the dsDNA amplicons and to release AuNPs into solution, where the electrochemical reduction of Au was measured by DPV. Combining the potential of RPA and surface-enhanced Raman scattering (SERS), the multiplex detection of three plant pathogens in the same reaction (not parallelised assays but real multiplex analysis) was achieved [33]. Four different tailed/biotinylated primers sets were used for RPA (one pair of primers for *B. cinerea*, one pair for *P. syringae*, one pair for *F. oxysporum* f. sp. *conglutinans* and another one for *F. oxysporum* f. sp. *lycopersici).* After amplification, RPA products were hybridised with AuNP-labelled probes incorporating different Raman reporters (4-mercaptobenzoic acid (MBA), 2,7-mercapto-4-methylcoumarin (MMC) and 2,3,5,6-tetrafluoro-4-mercaptobenzoic acid (TFMBA)) and captured on streptavidin-MBs. Upon laser excitation, specific Raman signals were generated corresponding to the specific plant pathogens (Figure 3). The entire assay required only 40 min and showed a sensitivity comparable to multiplex real-time PCR. The use of biotinylated and tailed primers allowed to perform the amplification in solution and the hybridisation of the amplicons with the capture and detector probes with no need of a denaturation step.

Otherwise, internal nucleic acid labelling was achieved by supplementing the RPA reaction mixture with labelled nucleotides. Labelled nucleotides such as biotin, digoxigenin or fluorescein-dUTPs are commercially available and randomly substitute dTTPs during polymerase extension to create labelled amplicons [69]. Ng and collaborators explored the use of biotin-labelled dUTPs for the development of an RPA-based electrochemical biosensor for *M. tuberculosis*. In one of the works, biotinylated amplicons were bound to streptavidin-AuNPs [34]. AuNPs were chemically oxidised by HCl to form the electrochemically active species AuCl_4_^-^, which was detected by DPV. In their next work, the biotinylated amplicons were bound to streptavidin-MBs and streptavidin-HRP [35]. The conjugates were incubated with TMB, whose electrochemical reduction was measured on screen-printed carbon electrodes. In both cases, LODs of 1 CFU were achieved. Compared to terminal labelling, internal labelling allows a higher number of labels to be incorporated into a single nucleic acid template.

Favourable thermal requirements, procedure simplicity and short amplification times make RPA process an advantageous technology for integration in biosensors. Although RPA is relatively new, the number of RPA-based biosensors is similar to those based on LAMP and RCA. Nevertheless, its application is still incipient, and more work is required for improving RPA performance and capabilities [70].

### 2.4. HDA-Based Biosensors

**Figure 4:**
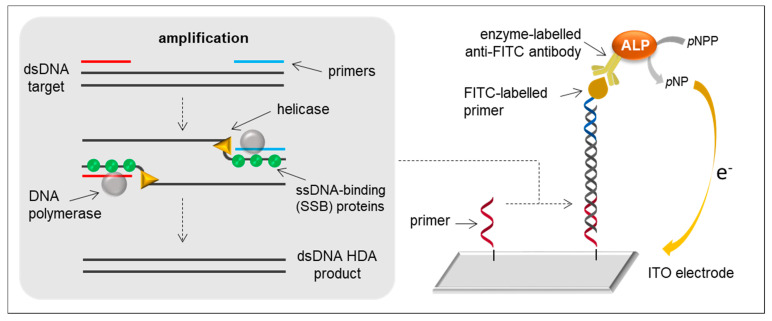
HDA principle and example of an HDA-based electrochemical biosensor using an immobilised primer, a FITC-labelled primer, an enzyme-labelled anti-FITC antibody and an ITO electrode.

HDA, first described in 2004 [71], makes use of a helicase enzyme instead of thermal treatment to dehybridise dsDNA. The process begins with helicase unwinding of dsDNA to which forward and reverse primers can bind, followed by polymerase-mediated elongation. After elongation, helicase can act again on the synthesised dsDNA and the cycle asynchronously repeats (Figure 4). HDA shows similar amplification kinetics to current PCR, but it is performed at a constant temperature of 60–65 °C for 60–120 min [42]. Nevertheless, depending on the biosensing strategy, a 95 °C strand denaturation step is sometimes required to enable subsequent amplicon hybridisation with the capture and/or reporter probe.

HDA was used for the amplification of *typA* [36] and *bipA* [37] genes of *Salmonella* spp. These genes are involved in the virulence and infectivity of the pathogen and are less subjected to spontaneous silent mutations than the gene *invA*, which most of the *Salmonella* spp. DNA amplification assays address. Although PCR primers may be used in HDA, they normally require more restrictive conditions (e.g., length between 24–33 bp), so they are normally modified to obtain the HDA ones [37]. In one of their works, Barreda-García and collaborators [36] immobilised thiolated primers to indium tin oxide (ITO) electrodes and solid-phase amplification took place. The ITO support was selected because of its improved thermal and storage stability compared to gold electrodes. A sandwich configuration was adopted using a FITC-labelled primer and an enzyme-labelled anti-FITC antibody. DPV was used to measure the 1-naphthol produced by the reaction of the enzyme alkaline phosphatase (ALP) with the substrate 1-naphthyl phosphate (Figure 4). In another work [37], the authors used a similar strategy but performed an exhaustive optimisation of several parameters, including the amount of capture probe immobilised on the electrode surface, the conditions of the immobilisation reaction, and the incorporation of a thermal treatment step before amplification to avoid secondary structures, among others. The HDA reaction was carried out in solution followed by a sandwich hybridisation assay using a capture probe immobilised on an ITO electrode and a FITC-labelled reporter probe for the subsequent interaction with the anti-FITC antibody-ALP conjugate. To allow hybridisation of the dsHDA product with the capture and reporter probes, amplicons were denatured. Denaturation rendered a ssHDA product that hybridised first with the reporter probe, which was present in the solution during the denaturation step, and afterwards with the immobilised capture probe. The electrochemical biosensors attained LODs of 2.4 nM of synthetic DNA for *typA* gene [36] and 10 copies of synthetic DNA for *bipA* gene [37] of *Salmonella* spp.

HDA in solution was also used in the development of biosensors for *M. tuberculosis*. Torres-Chavolla and Alocilja [38] used amine-terminated MBs as capture probe immobilisation supports and AuNP-labelled reporter probes. After hybridisation with HDA amplicons previously dehybridised, the MB-target-AuNP complexes were immobilised on the electrode using a magnetic support and oxidation of AuNPs was monitored electrochemically by DPV. Barreda-García and co-workers [39] also used MBs as immobilisation supports coupled with liquid-phase HDA. In this case, they used a biotinylated primer and streptavidin-coated MBs, which were immobilised on a magnetised screen-printed carbon electrode. In this work, to avoid denaturation of the dsDNA products, an asymmetric HDA (AHDA) reaction was performed. By using one of the primers in excess (in this case, the biotinylated one), ssHDA products were generated, thus enabling their hybridisation with a FITC-labelled reporter probe, finally detected through an anti-FITC-HRP conjugate. The biosensor attained an LOD of 0.5 aM of synthetic DNA for *M. tuberculosis.*

The protocol time of HDA has been considered a limitation when comparing with other isothermal DNA amplification methods. Nevertheless, the simplicity of the two-primer reaction allows a faster optimisation. Only two HDA-based electrochemical biosensors for the detection of *Salmonella* spp. and *M. tuberculosis* have been developed. The higher reaction temperatures compared with other isothermal methods make HDA less prone to non-specific amplification attributable to mispriming as a consequence of a constant temperature amplification.

### 2.5. SDA-Based Biosensors

**Figure 5:**
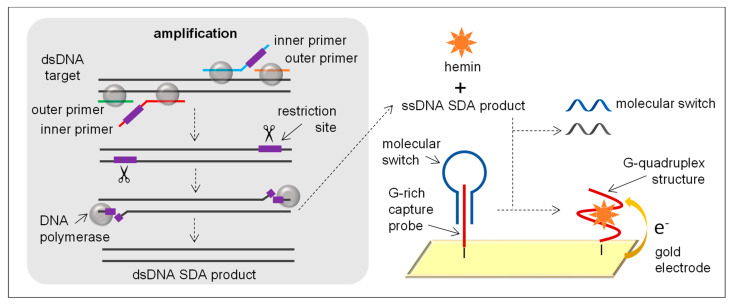
SDA principle and example of an SDA-based electrochemical biosensor using a G-rich capture probe, a molecular switch, hemin and a gold electrode.

SDA relies on the use of four primers: two outer primers and two inner primers. Inner primers contain a sequence complementary to the target and a sequence for nicking endonuclease at the 5′. Outer primers are located upstream of the inner primers and are complementary to the target sequence. These primers bind to the target and are extended by specific DNA polymerases such as exo- Klenow or *Bst* with strand displacement activity. Upon the formation of strands harbouring a nicking site (linear amplification), the nicking enzyme nicks them and the DNA polymerase starts a new round of replication (Figure 5). Exponential amplification of the target sequence starts by cycle of single and double nicking, extension, and displacement. The complex asynchronous reactions occur concurrently at 37 °C (after initial heat denaturation for dsDNA targets).

Although SDA was first described in 1992 [72], to the best of our knowledge, only one SDA-based biosensor for the detection of *S. aureus* has been published [40]. In this work, an aptamer is used as a biorecognition molecule. Aptamers are oligonucleotide or peptide molecules that can selectively bind to a specific target, including proteins, peptides, carbohydrates, small molecules, toxins and even live cells [73]. A biotinylated aptamer bound to a complementary ssDNA sequence was immobilised on streptavidin-modified MBs. When *S. aureus* was present, the aptamer bound to *S. aureus* and released the complementary ssDNA to the solution, which was amplified by means of SDA. Although the final product of SDA is dsDNA, ssDNA is generated during the amplification process. This ssDNA was detected using gold electrodes modified with molecular switches fastened to G-rich capture probes. The loop of molecular switches was complementary to the amplified ssDNA, so in the presence of the amplified ssDNA the molecular switch was leaked from the electrode and the capture probes were released to react with hemin to form an electrically active G-quadruplex structure that turned on the electrochemical signal (Figure 5). The biosensor showed an LOD of 8 CFU/mL and was applied to the analysis of spiked lake water, tap water and honey samples, achieving recoveries of 93.9–101.8%. Since the molecular switch used in the SDA-based biosensor was not related to the aptamer sequence, the same strategy was applied to the detection of *E. coli* by changing the aptamer, the terminals sequence of the complementary ssDNA and the primers. In this case, an LOD of 22 CFU/mL was achieved.

In the work performed by Cai and co-workers [40], the amplification step was performed for 45 min, which is a shorter time compared with the original 2-h SDA process which has been considered too long for the development of biosensors. Moreover, SDA reactions have shown high sensitivity to background DNA, which will coamplify following non-specific primer binding as a result of reduced stringency conditions present at 37 °C. The use of aptamers provides high selectivity to the assay ascribed to the high specificity of the aptamers for their corresponding targets.

### 2.6. ISDPR-Based Biosensors

**Figure 6:**
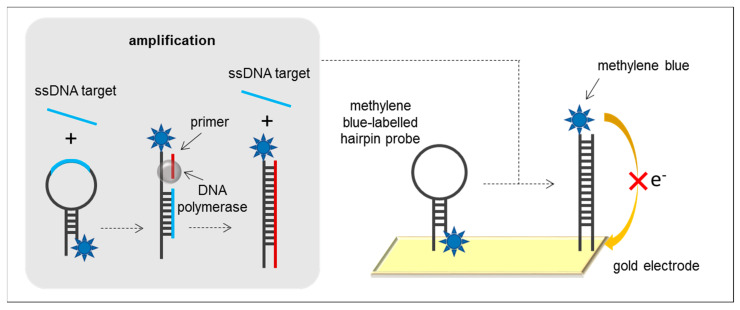
ISDPR principle and example of an ISDPR-based electrochemical biosensor using a methylene blue-labelled hairpin probe and a gold electrode.

ISDPR is a new member of the family of isothermal amplification techniques, first introduced in 2009 by Guo et al. [74]. This method uses a hairpin DNA probe, a short primer, and a DNA polymerase. While the hairpin loop sequence is complementary to the target sequence, the 3′-end of the probe is complementary to the primer. In the absence of the target, the hairpin probe is unable to anneal with the primer. In the presence of the target, the hairpin probe undergoes a conformational change, leading to the stem separation because of the probe-target hybridisation. After this, the primer anneals with the open stem and induces a polymerisation reaction in the presence of both DNA polymerase I (exo- Klenow fragment) and dNTPs, leading to primer extension and target displacement. The displaced target recognises and hybridises with another probe, initiating the next round of polymerisation reaction (Figure 6). ISDPR is commonly carried out for 120 min at 37 °C, but usually requires an initial denaturation step at 95 °C to generate ssDNA able to hybridise with the hairpin probe.

The original method relied on a hairpin fluorescence probe carrying a fluorophore and a quencher moiety linked to the ends of the stem. The hairpin conformational change in the presence of the target resulted in the separation of the quencher from the fluorophore and the subsequent generation of a fluorescent signal. Wang and collaborators [41] used methylene blue-labelled hairpin probes self-assembled on gold electrodes for the detection of *mecA* gene of *S. aureus*. In the absence of the target, the redox moieties were close to the electrode surface, resulting in an efficient electron transfer. The hybridisation of *mecA* gene with the hairpin probe underwent a conformational change that moved methylene blue molecules away from the electrode surface and resulted in a decrease of the current response (Figure 6). Current reduction increased as the target strand was replaced by the amplified strand and it was hybridised to another hairpin probe, achieving an LOD of 63 fM of synthetic DNA and a wide linear range from 0.075 to 200 pM.

Only one electrochemical ISDPR-based biosensor has been developed for the detection of bacteria, probably due to the relative novelty of the amplification method, and it was only applied to the detection of synthetic DNA and not to genomic DNA. ISDPR requires an initial heating step for dsDNA targets and results in linear amplification, which leads to lower amplification yields compared to exponential amplification techniques. Moreover, the length of the DNA target plays a crucial role in this amplification technique, as it requires to be hybridised with the hairpin probe. The use of a labelled-hairpin probe provides a singular detection strategy without the need of additional reporter probes.

## 3. Isothermal DNA Amplification-Based Biosensors: Key Factors

Biosensors harness the specificity and sensitivity of biological systems in small, low-cost devices providing a powerful alternative to conventional methods for rapid and on-site detection of bacterial contamination. Detection of specific DNA sequences enables highly specific typing of bacteria. To ensure an appropriate sensitivity, a DNA amplification step is required. The use of isothermal DNA amplification methods, which operate at a constant temperature, simplifies the development of integrated and portable devices. Since usually enzymes are used to perform strand separation, repeated heating (thermal cycling), required in PCR, is avoided. In the development of such biosensors, each isothermal DNA amplification method has pros and cons to be considered, which are summarised in Table 2. A fair comparison of the performance of isothermal DNA amplification-based biosensors is difficult to make due to the multitude of variables that must be considered (target type, sample pre-concentration, detection strategy, among others), in addition to the differential metrics used for performance evaluation. Nevertheless, in what follows, the key factors are discussed with the purpose to provide critical analysis of the state of the art and the challenges that need to be tackled.

### 3.1. Operating Temperature

The first important consideration regarding the integration of isothermal DNA amplification technologies in biosensors is the operating temperature. The reaction temperatures range between 37 °C and 65 °C. Higher temperatures increase power demand, but this drawback is countered by an increase in the reaction kinetics as well as in the stringency and the specificity of the amplification reaction due to the reduction of non-specific primer annealing. Otherwise, as with PCR, many isothermal techniques (e.g., RCA, SDA and ISDPR) require an initial heating step (~95 °C) to dehybridise the target dsDNA. This additional heating step complicates control mechanisms and further increase the power demand. RPA and HDA techniques can act directly on dsDNA without a heating step, which is of great interest when using isothermal strategies. LAMP neither requires an initial denaturation step, but it has been demonstrated that a heating step prior amplification usually enhances sensitivity [75]. Moreover, some isothermal methods such as LAMP and RCA can include a final step after amplification at 80 °C or 60–95 °C, respectively, for few minutes to increase assay sensitivity [16,25,26]. Additionally, it is important to keep in mind that depending on the detection strategy, an additional heating step to allow the hybridisation of the dsDNA amplicon with the capture and/or reporter probe can be needed.

### 3.2. Assay Design

The assay design also needs to be considered. Similarly to PCR, HDA and RPA methods rely on two primers, which may facilitate the integration of such methods in biosensors. However, other methods such as LAMP require up to six primers. The use of additional primers complicates the design but improves the specificity of the assay and can increase the speed reaction, as observed with the use of additional loop primers in LAMP. In the case of RCA, the use of PLPs to amplify linear DNA targets results in a higher specificity of the assay but increases the time and complexity of the system.

Multiplex amplification is pursued in most strategies to allow detection of multiple targets and discrimination between pathogens at the same time. Unfortunately, as happens with PCR, multiplexing of isothermal amplification reactions results in a highly complex interplay of factors including primer competition and interaction, amplification bias and product interactions. Isothermal methods with simpler designs are more easily adaptable to multiplex detection. Nevertheless, only a triplex SERS assay using RPA as an isothermal amplification method has been developed for the single-tube multiplex detection of three bacterial plant pathogens [33]. Further increasing multiplexing level is very challenging when performing single-tube multiplex. Some other works have performed multiplex detection using parallelised amplifications, i.e., performing multiple single reactions next to each other in an independent way. Parallelised multiplex amplification circumvents cross-reaction issues and possesses higher multiplex capacity compared to the single-tube multiplex RPA, being only limited by the detection method.

### 3.3. Type of Analyte

Another crucial consideration is the type of analyte to be detected, which can be synthetic DNA, genomic DNA or CFU. Most of the biosensors based on isothermal DNA amplification methods employ synthetic DNA to demonstrate the feasibility of the biosensor, since it is the simplest target to work with. Although working with genomic DNA is more complex, it represents the target present in nature and thus is much closer to reality. Even more demanding is working with CFUs, since it provides an estimation of the viable bacteria cells. It is a very good option to compare results with traditional techniques like cell culture, which uses this unit. Nevertheless, when detecting DNA it is not possible to discriminate between viable and non-viable bacteria in real samples. One possible solution is the use of specific DNA intercalating dyes such as propidium monoazide (PMA) [76]. This compound can penetrate dead cells but not into live cells with intact cell membranes. After intercalation into DNA, PMA treatment renders the DNA insoluble, resulting in its separation during genomic DNA extraction. Therefore, PMA treatment allows obtaining signals from only live bacterial cells.

Besides the type of analyte, it is also important to consider the type of target. This can be dsDNA or ssDNA and linear or circular. While some isothermal DNA amplification techniques can act either on dsDNA or ssDNA targets (LAMP, RPA and HDA), some others only amplify ssDNA (RCA, SDA and ISDPR), thus a denaturation step before amplification being a pre-requisite. Moreover, in contrast to LAMP, RPA, HDA, SDA and ISDPR, which are able to amplify only linear DNA, RCA is able to amplify both linear and circular DNA.

### 3.4. Detection Strategy

When designing the detection strategy, the type of amplification product generated should be considered. The products of the methods outlined herein can be ssDNA or dsDNA and some of them adopt a concatenated configuration (LAMP and RCA). Thus, the detection strategy to be used has to be compatible with those products and will be selected according to the requirements that the biosensor needs to accomplish. While electrochemical methods provide advantages in terms of simplicity, miniaturisation and low cost, which make them ideal for in situ detection, optical biosensors present high sensitivity and reliability and allow label-free and multi-analyte detection.

DNA amplification can be measured after the reaction (endpoint detection) or while the reaction is progressing (real-time detection). Endpoint detection requires less complex instrumentation and provides outputs simpler to be interpreted. Real-time methods integrate amplification with detection and can provide more reliable quantitative results. In all cases, detection methods able to differentiate target-specific amplification from non-specific amplification products and that minimise the risk of carry-over contamination are desired.

### 3.5. Applicability

Finally, the application of the biosensors to the analysis of real samples is of utmost importance. While current molecular diagnostic technologies require high purity DNA samples, isothermal amplification techniques are usually more tolerant to enzyme inhibitors present in real samples, showing equivalent or even higher sensitivity than thermal cycling amplification [77]. Nevertheless, the application of biosensors based on isothermal DNA amplification methods to the analysis of bacteria in real samples is still incipient, and only a few of them have been applied in the fields of food safety and environmental monitoring. LAMP was used for the detection of *Y. enterocolitica* in pork meat using an electrochemical biosensor [16] and for the detection of *E. coli* 0157:H7 in spiked apple juice and milk samples with a fiber optic sensor [19]. LAMP-based electrochemical biosensors were also used to detect *E. coli* and *S. enterica* in juice, milk and soy milk [47] and a LOC platform device using SAW detection was applied to the detection of *S.* Typhimurium in milk [20]. An RCA-based electrochemical biosensor was used for *C. tetani* detection in soil samples [24]. RPA-based electrochemical biosensors were used for the detection of *P. salmonis* in salmon [28], *F. tularensis* in hares [29] and *P. syringae* in *Arabidopsis* plants [32]. Moreover, *Arabidopsis* and tomato plants were analysed by a multiplexed RPA-based SERS platform [33] for the detection of *P. syringae*, *B. cinerea* and *F. oxyspurim.* Finally, the SDA-based biosensor was applied to the detection of *E. coli* in spiked lake water, tap water and honey samples [40]. Some of the mentioned works showed good recovery values [19,24,40] and correlated well with results obtained with other techniques such as qPCR [28,32] or PCR and gel electrophoresis [20,29,33]. Nevertheless, a more exhaustive work is required before implementation of these analytical tools. Matrix effects are complex and system-specific and should be addressed prior to the application of biosensors to the analysis of natural samples. Furthermore, the results obtained need to be compared with other analytical methods such as PCR or qPCR or traditional cell culture, and the different recognition principles between techniques should be considered to understand the correlation between the obtained results. The analysis of a high number of naturally contaminated samples is also required for the validation of the biosensors. Even so, these preliminary results show the potential application of biosensors based on isothermal DNA amplification for the detection of multiple bacteria pathogens in different matrix samples for numerous purposes.

### 3.6. Point-of-Need Testing

When implementing analytical devices, the whole analysis process (including sampling, sample pre-concentration, nucleic acid extraction, amplification and detection) needs to be considered. Recently, a significant demand and effort in merging biosensors into LOC technology using microfluidics systems has been observed, with the aim to integrate sample preparation steps and analytical processes in a single miniaturised device [78].

Extraction of DNA is a crucial step for successful pathogen detection by a molecular method, since the reproducibility and sensitivity of the detection directly depends on the integrity and purity of the DNA. Bacterial cells need to be liberated from the matrix, followed by the lysis of the cells by disruption of their walls. Usually, a DNA purification step is required to separate DNA from proteins and cell debris, although isothermal amplification methods have been shown to be more tolerant to matrix effects than thermal cycling.

The efficiency of the DNA extraction method can be improved with a cell pre-concentration step before the extraction. Antibodies, aptamers or bacteriophage-derived proteins can be used for capture, isolation and enrichment of the given pathogen prior to analysis [79]. The use of functionalised MBs can help to miniaturise and automate these pre-concentration protocols to be integrated into microfluidic devices. This pre-concentration step is particularly important for bacterial detection in LOC devices as it increases bacterial concentration and reduces the volume to analyse.

It is important to take in mind that in LOC and microfluidic devices the volumes handled must be kept as low as possible. At such small volumes, the target DNA content will be very low. Thus, a DNA amplification step is crucial to provide the biosensor with enough sensitivity. The use of isothermal DNA amplification methods has gained popularity due to the reduction in power consumption and compatibility with miniaturisation, being easier to operate and providing equivalent sensitivity than thermal cycling amplification.

The proper combination of sample preparation, amplification and detection facilitates the development of fully integrated point-of-need analytical devices, easy to be operated by consumers. The availability of cost-effective, user-friendly readers that interpret the signal from the biosensor and provide clear results to the end user will be the key to implement LOC devices far from the laboratory bench. In this sense, the incorporation of biosensing devices into smartphone platforms is opening the door to a much broader range of users to perform in situ analysis [80]. Although technical obstacles related to achieving appropriate integration of multiple crucial components in a robust, user-friendly format, while minimising complexity and cost are still remaining, we expect that these sensing modalities will become soon available, offering new opportunities for enhancing food safety and environmental monitoring.

## 4. Conclusions and Future Perspectives

Despite the great potential of isothermal DNA amplification methods and biosensing strategies, the application of such biosensors to bacterial detection is still in its infancy. Efforts and time are needed to replace, or at least, live together with traditional laboratory methods based on bacterial culture and thermal cycling amplification. In our humble opinion, the lack or small number of companies that provide ready-to-use kits to carry out some of the isothermal amplification techniques hinders the subsequent development of biosensors. Furthermore, implementation of isothermal DNA amplification-based biosensors in routine monitoring programs still requires advances in sample processing and DNA purification, together with validation of the systems using a high number of food and environmental samples. Nevertheless, having in mind the benefits provided by the integration of isothermal DNA amplification in biosensors and the wide possibilities that the different detection strategies afford, hope lies in better accessibility of these molecular technologies and a subsequent effort to cover the entire analysis process from sample processing to signal readout. This will clearly provide powerful analytical tools with high sensitivity and specificity, rapidity, ease of use, low cost, automation and portability, which will revolution food safety and environmental monitoring.

## Figures and Tables

**Table 1:** Overview of bacterial biosensors based on isothermal DNA amplification methods: LAMP, RCA, RPA, HAD, SDA and ISDPR-based methods.

Isothermal Amplification Method	Bacteria	Target Gene	Amplification System	Transducer Functionalisation	Detection Strategy	Detection Method	LOD	Applicability	Ref.
LAMP	*S. aureus*	*femB* *femA*	In solution	Biotin-capture probes on a streptavidin-modified SPR chip	AuNP-reporter probe	Optical (SPR)	10 copies/µL genomic DNA	Clinical samples (blood and sputum)	[14]
LAMP	*V. cholerae*	*lolB*	In solution	Capture probe electrodeposited on a screen-printed carbon electrode	AuNP-PSA/avidin-reporter probe	Electrochemical (DPASV)	50 ng/µL genomic DNA	-	[15]
LAMP	*Y. enterocolitica*	*gyrB*	In solution	Capture probe immobilised on a chitosan-nano-V_2_O_5_-MWCNT-modified carbon paste electrode	Methylene blue intercalation into the amplicon hybridised to the capture probe	Electrochemical (DPV)	1.76 fM synthetic DNA	Pork meat	[16]
LAMP	*S.* Typhimurium *S.* Choleraesuis	*recF*	In solution	Methylene blue-capture probes self-assembled on a gold electrode	Methylene blue-capture probes conformational change produced by target hybridisation	Electrochemical (SWV)	350 CFU/mL	Clinical samples (blood)	[17]
LAMP	*S. aureus*	PVL toxin	In solution	Non-functionalised tuneable fluidic nanopore sensor	Biotin-primer and avidin-HRP, and AuNP-primer	Electrochemical (RPS)	530 copies genomic DNA	-	[18]
LAMP	*E. coli*	-	In solution	Anti-E-coli polyclonal antibody immobilised on a CNT multilayer-modified ITO electrode	Monitoring of the turbidity of the magnesium pyrophosphate white precipitate produced during amplification	Optical (fiber optic sensor)	1 CFU/mL	Spiked apple juice and milk	[19]
LAMP	*S.* Typhimurium	*invA*	In solution	Anti-*S.* Typhymurium antibody immobilised on an oxygen plasma nanotextured polymeric chip	Monitoring changes in the acoustic wave energy	Optical (SAW)	1 CFU/25 mL	Milk	[20]
RCA	*M. tuberculosis* *M. avium*	-	In solution	Capture probes self-assembled on an AuNP-modified SPR chip	Amplicon cleavage and hybridisation with the capture probes	Optical (SPR)	4.2 × 10^4^ CFU/mL and 5 pg/µL genomic DNA for *M. tuberculosis* 3.7 × 10^4^ CFU/mL and 2 pg/µL genomic DNA for *M. avium*	Clinical samples (sputum, urine and cerebrospinal fluid)	[21]
RCA	*M. tuberculosis*	-	Solid phase	Primer self-assembled on an SPR chip	AuNP-reporter probe	Optical (SPR)	5 pM synthetic DNA and 8.2 pg/µL genomic DNA	Clinical samples (sputum, urine and clinical isolates)	[22]
RCA	*E. coli* *E. faecalis* *S. dysenteriae* *S. pneumoniae* *S. epidermidis* *S. aureus*	-	Solid phase	Biotin-primers on streptavidin-AuNPs bound to biotin-secondary antibodies immobilised on an SPR chip	AuNP-reporter probes	Optical (SPR)	0.5 pM synthetic DNA and 0.5 pg/µL genomic DNA	Clinical samples (sputum, urine and faeces)	[23]
RCA	*C. tetani*	Tetanus toxin	In solution	Capture probe on an AuNP-MWCNT-modified glassy carbon electrode	Methylene blue-reporter probe	Electrochemical (DPV)	1 fM synthetic DNA	Soil	[24]
RCA	*V. cholerae*	-	In solution	-	MNP-reporter probe and monitoring of the MNP aggregation	Optical (FMR)	1 pM synthetic DNA	-	[25]
RCA	*E. coli*	-	Solid phase	Biotin-primer on streptavidin-MBs	Magnetic separation of the amplicon	Optical (THz spectroscopy)	120 pM synthetic DNA and 50 pg/µL genomic DNA	-	[26]
HRCA	*L. monocytogenes*	*hly*	Solid phase	Biotin-primer on streptavidin-MBs magnetised on a platinum electrode	TBR-primer	Optical (electrochemiluminescence)	10 aM synthetic DNA and 0.2 pg/µL genomic DNA	Milk	[27]
RPA	*P. salmonis*	-	Solid phase	Primer self-assembled on a gold electrode	Biotin-primer and streptavidin-HRP	Electrochemical (amperometry)	50 ag/µL synthetic DNA and 3 × 10^2^ copies/µL genomic DNA	Salmon	[28]
RPA	*F. tularensis*	-	Solid phase	Primer self-assembled on a gold electrode	Biotin-primer and streptavidin-HRP or HRP-primer	Electrochemical (amperometry)	5.3 fM synthetic DNA and 500 fM copies genomic DNA	Hares	[29]
RPA	*F. tularensis*	-	Solid phase	Primer on an azido-modified ring resonator chip	Label-free detection	Optical (ring resonator)	78 pM synthetic DNA	-	[30]
RPA	*Salmonella* spp.	*bipA*	Solid phase	Primer self-assembled on a gold electrode	FITC-primer and anti-FITC antibody-HRP	Electrochemical (amperometry)	10^5^ copies genomic DNA	-	[31]
RPA	*P. syringae*	-	In solution	Biotin-primer on streptavidin-MBs	AuNP-reporter probe	Electrochemical (DPV)	214 pM genomic DNA	*Arabidopsis* plants	[32]
RPA	*P. syringae* *B. cinerea* *F. oxysporum*	-	In solution	Biotin-primers on streptavidin-MBs	AuNP-reporter probes complementary to the tailed-primers with different Raman reporters	Optical (SERS)	2 copies genomic DNA	*Arabidopsis* and tomato plants	[33]
RPA	*M. tuberculosis*	-	In solution	Non-functionalised screen-printed carbon electrode	Biotin-dUTP amplicon and streptavidin-AuNPs	Electrochemical (DPV)	1 CFU/mL	-	[34]
RPA	*M. tuberculosis*	-	In solution	Non-functionalised screen-printed carbon electrode	Biotin-dUTP amplicon and streptavidin-HRP	Electrochemical (amperometry)	1 CFU/mL	-	[35]
HDA	*Salmonella* spp.	*typA*	Solid phase	Primer on an ITO electrode	FITC-primer and anti-FITC antibody-ALP	Electrochemical (DPV)	10 copies synthetic DNA	-	[36]
HDA	*Salmonella* spp.	*bipA*	In solution	Capture probe on an ITO electrode	FITC-reporter probe and anti-FITC antibody-ALP	Electrochemical (DPV)	50 copies synthetic DNA	-	[37]
HDA	*M. tuberculosis*	*IS6110*	In solution	Capture probe on amine-MBs	AuNP-reporter probe	Electrochemical (DPV)	10 pg/µL synthetic DNA	-	[38]
AHDA	*M. tuberculosis*	-	In solution	Biotin-primer on streptavidin-MBs magnetised on a screen-printed carbon electrode	FITC-reporter probe and anti-FITC antibody-HRP	Electrochemical (amperometry)	0.5 aM synthetic DNA	Clinical samples (sputum, pleural fluid and urine)	[39]
SDA	*S. aureus* *E. coli*	*-*	In solution	Molecular switch fastened to G-rich capture probe on a gold electrode	Molecular switch displacement by the amplicon and formation of electroactive G-quadruplex/hemin complex	Electrochemical (DPV)	8 CFU *S. aureus*/mL 22 CFU *E. coli*/mL	Lake water, tap water and honey	[40]
ISDPR	*S. aureus*	*mecA*	Solid phase	Methylene blue-hairpin probe self-assembled on a gold electrode	Methylene blue-hairpin probe conformational change produced by target hybridisation	Electrochemical (SWV)	63 fM synthetic DNA	-	[41]

**Table 2:** Characteristics of the isothermal DNA amplification methods: LAMP, RCA, RPA, HDA and ISDPR.

	LAMP	RCA	RPA	HDA	SDA	ISDPR
Target type	dsDNA and ssDNA, linear	dsDNA and ssDNA, circular and linear	dsDNA and ssDNA, linear	dsDNA and ssDNA, linear	dsDNA and ssDNA, linear	dsDNA and ssDNA, linear
Steps prior amplification	Heating at 95 °C for dsDNA targets (optional)	Heating at 95 °C for dsDNA targets; PLP ligation and exonuclease treatment at 37–95 °C for linear target	-	-	Heating at 95 °C for dsDNA targets	Heating at 95 °C for dsDNA targets
Amplification type	Exponential	Linear (RCA) and exponential (HRCA)	Exponential	Exponential	Exponential	Linear
Amplification temperature	60–65 °C	37–40 °C	37–40 °C	60–65 °C	37 °C	37 °C
Number of primers	4–6	1–2 + PLP probe for linear targets	2	2	4	1 + hairpin probe
Overall process time	~60 min	~150 min	20–40 min	60–120 min	45–120 min	~120 min
Steps after amplification	Heating at 80°C	Heating at 60°C	-	-	-	-
Product type	dsDNA or ssDNA, concatenated with loop configuration	dsDNA (HRCA) or ssDNA (RCA), concatenated with linear configuration	dsDNA, linear	dsDNA, linear	dsDNA or ssDNA, linear	dsDNA, linear

## Data Availability

Not applicable.
